# Design of Real-Time Demodulation for FBG Sensing Signals Based on All-Dielectric Subwavelength Gratings Edge Filters

**DOI:** 10.3390/nano15070536

**Published:** 2025-04-01

**Authors:** Jingliang Lin, Ping Tang, Kaihao Chen, Jiancai Xue, Ziming Meng, Jinyun Zhou

**Affiliations:** 1 School of Physics and Optoelectronic Engineering, Guangdong University of Technology, Guangzhou 510006, China; 2112215055@mail2.gdut.edu.cn (J.L.);; 2 Key Laboratory of Photonics Technology for Integrated Sensing and Communication of Ministry of Education, Guangdong University of Technology, Guangzhou 510006, China

**Keywords:** sapphire fiber Bragg gratings, high-temperature sensing, all-dielectric subwavelength grating, wide-range demodulation, Mie resonance modes

## Abstract

Accurate real-time temperature measurement under extreme thermal-pressure conditions remains challenging in aerospace. Sapphire fiber Bragg gratings (FBGs), exhibiting temperature measurement capabilities up to 1900 °C, demonstrate suitability for such extreme environments. However, the development of a high-performance demodulation system capable of processing sapphire FBG signals over wide spectral ranges at elevated speeds remains a technical challenge. This study presents a real-time FBG signal demodulation system that incorporates an all-dielectric subwavelength grating edge filter. The designed grating, comprising a TiO_2_/Si_3_N_4_ subwavelength unit array, modulates Mie-type electric and magnetic multipole resonances to achieve precisely tailored transmission and reflection spectra. Simulation results indicate that the grating exhibits low ohmic loss, excellent linearity, complementary transmission/reflection characteristics, a wide linear range, and angular-dependent tunability. The designed edge-filter-based demodulation system incorporates dual single-point detectors to simultaneously monitor the transmitted and reflected signals. Leveraging the functional relationship between the center wavelength of the FBG and the detected signals, this system enables high-speed, wide-range interrogation of the center wavelength, thus facilitating real-time demodulation for wide-range temperature sensing. The proposed method and system are validated through theoretical modeling, offering an innovative approach for sapphire FBG signal demodulation under extreme thermal-pressure conditions.

## 1. Introduction

Accurate temperature sensing under high-temperature and high-pressure conditions remains a critical challenge for aerospace applications. A representative case involves next-generation high-thrust aircraft engines, whose operational safety demands continuous temperature tracking with second latency [1,2]. In such extreme environments, conventional thermocouple-based measurement methods, which rely on precious metal thermocouple temperature sensors, encounter significant limitations. These sensors cannot maintain stable operation over extended periods and are unsuitable for distributed temperature measurement in enclosed furnace environments [3]. Comparatively, fiber Bragg grating (FBG) sensors exhibit multiple superior characteristics: high-temperature endurance, electromagnetic interference rejection capability, robust durability, compact size, wide operational temperature spans, and excellent multiplexing capabilities. These properties render FBG sensors highly promising for temperature sensing in extreme environments [4]. The refractive index of the FBG core exhibits periodic and uniform distribution, effectively functioning as a narrowband filter. When broadband light propagates to the FBG, the incident wavelength satisfying the Bragg condition is reflected while other wavelengths are transmitted. Variations in external temperature alter the periodic structure of the FBG, thereby modifying the Bragg condition and inducing a shift in the Bragg wavelength of the reflected light [5]. This unique characteristic of FBGs renders them particularly effective for temperature detection. Notably, sapphire FBGs, fabricated via femtosecond laser inscription technology in multimode single-crystal sapphire fibers, can achieve temperature sensing up to 1900 °C [6], meeting the demanding requirements of the aerospace industry requiring temperature measurement under high-temperature and high-pressure conditions.

However, despite the sapphire FBG sensors are capable of withstanding ultra-high temperature measurements, their high-temperature operational range is accompanied by significant Bragg wavelength drift across a wide range [6]. Additionally, rapidly fluctuating environmental temperatures necessitate a demodulation system with ultrafast response speeds. Consequently, there is an urgent demand for FBG signal demodulation systems that feature wide demodulation ranges and high demodulation rates [7]. One rapid and cost-effective demodulation approach is the edge filtering method based on wavelength edge filters. In this method, the edge filter functions as an optical filter that exploits differences in transmittance and reflectance for distinct wavelengths, converting the wavelength shifts of the FBG into measurable variations in optical power intensity. This enables extremely high demodulation speeds, permitting real-time monitoring of temperature variations. By optimizing the performance of the optical filter and expanding the linear edge filtering range, this method can be effectively applied to the demodulation of high-temperature sapphire FBG sensors [8].

The diverse selection of edge filters has led to multiple implementations of the edge filtering method. One of the earliest approaches, the bulk filter method [9], utilizes volumetric optical filtering elements as the filters. However, the measurement accuracy of this method is significantly affected by the alignment and stability of the filtering components, resulting in lower resolution, bulky design, and poor portability. Replacing bulk optical filters with fiber-optic wavelength-division multiplexing (WDM) couplers as linear filters offers an alternative solution [10]. This approach leverages the approximately linear relationship between transmittance and wavelength at the two ports of the WDM, enabling the realization of an all-fiber linear demodulation system. Nevertheless, the polarization-dependent characteristics of WDM limit the precision of wavelength measurements. Another method employs the logarithmic ratio of adjacent channel intensities in arrayed waveguide gratings (AWGs), which exhibits a linear relationship with the wavelength of FBGs under Gaussian approximation [11]. This technique is suitable for applications requiring high-precision strain and temperature measurements. However, the limited channel bandwidth of AWGs restricts the demodulation system to achieving high wavelength resolution only within a narrow dynamic range [12].

**Figure 1:**
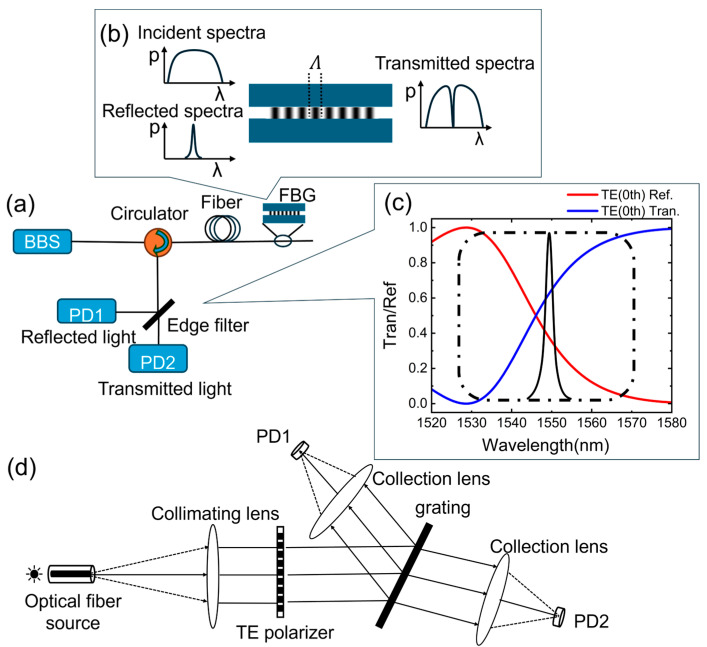
Schematic diagram of the demodulation principle. (**a**) A broadband source (BBS) emits light, which is directed through a circulator to the FBG. Only light matching the Bragg wavelength is reflected onto the all-dielectric metasurface grating edge filter; (**b**) working principle of the fiber Bragg grating; (**c**) the operational wavelength range of the FBG (dashed box) corresponds to a region where the wavelength dependence of the ratio approximates a linear function; (**d**) the reflected and transmitted beams are captured by dual single-point detectors, with their respective optical power levels recorded as PD1 and PD2.

Camilo et al. employed a catastrophic fuse-effect microcavity Fabry–Pérot (F–P) interferometer as an edge filter [13]. While this approach delivers high resolution, its slow demodulation speed renders it unsuitable for high-speed applications. Alternative edge filters include reflective matched FBGs, long-period gratings (LPGs), and chirped fiber Bragg gratings (CFBGs). Reflective matched FBGs achieve exceptionally high resolution but are constrained by a narrow dynamic range [14]. LPGs enable measurement of large temperature variations, albeit at the expense of reduced temperature resolution [15]. CFBGs exploit the linear rising or falling edges of their reflection spectra as edge filters [16], allowing adjustable demodulation ranges based on the linear region. However, operating outside this linear range introduces significant errors. Ken et al. utilized the linear dependence of the transmission/reflection ratio on wavelength within the transition band between the stopband and passband of an optical filter to implement edge filtering [17]. By optimizing output configurations, this method mitigates the impact of signal source fluctuations, thereby enhancing the reliability of intensity measurements. Nevertheless, its narrow demodulation range restricts broader applicability. In this study, we designed an all-dielectric subwavelength grating as an edge filter to realize the edge filtering demodulation method. Figure 1 illustrates the schematic diagram of the demodulation principle.

The all-dielectric subwavelength grating falls under the classification of all-dielectric metasurfaces—a class of two-dimensional (2D) metamaterials comprising subwavelength-scale structural arrays fabricated from dielectric materials such as titanium dioxide (TiO_2_), silicon (Si), gallium arsenide (GaAs), and silicon nitride (Si_3_N_4_). By leveraging Mie resonance theory, these structures support both electric and magnetic multipole modes, offering advantages including high refractive indices, minimal ohmic losses, and cost efficiency. Consequently, they are widely employed in the design and realization of high-performance optical devices [18]. Metasurfaces enable precise manipulation of electromagnetic wave properties—such as wavelength, amplitude, phase, and polarization—through strategic material selection and spatially tailored structural configurations. Rational design methodologies have facilitated the realization of metasurface grating structures exhibiting edge filtering effects [19,20,21]. In this study, we propose replacing conventional diffraction grating in spectrometers with our all-dielectric metasurface grating. When integrated with dual single-point detectors, this configuration achieves high-speed real-time demodulation of FBG signals, overcoming the limitations of traditional spectral analysis systems.

The contributions of this work are summarized as follows: First, we employed the finite element method (FEM) to optimize the geometry and parameters of the all-dielectric grating. Diffraction curves of the grating were calculated to achieve tailored edge filtering performance. Second, mechanistic analysis of electromagnetic interactions: the electromagnetic field distributions across wavelengths within the grating’s operational range were analyzed to elucidate the physical mechanisms underlying the edge-filtering effect. Furthermore, the influence of critical geometric parameters on the grating’s performance was investigated, establishing design rules for performance tuning. Finally, the feasibility of this demodulation method for high-speed and wide-range demodulation of sapphire FBG signals was validated. The results demonstrate that the operational wavelength range of the all-dielectric metasurface grating can be dynamically adjusted through targeted parameter modifications, underscoring its practical application value.

## 2. Methods

The subwavelength grating-based edge filter, designed for FBG sensors demodulation, operates across a wavelength range of 1535–1580 nm. The diffraction orders of the reflected light are governed by the grating equation [22]:

(1) $$n_{0}sin\theta_{L}-n_{0}sin\theta =\frac{L\lambda }{d},$$

where L represents the diffraction order,$\;\theta_{L}$ is the diffraction angle of the Lth order, $n_{0}=1.00$ is the refractive index of the air environment, λ is the wavelength of the incident light, and d is the grating period. Because the sine functions can only vary between −1 and 1, the existence of higher diffraction order requires that

(2) $$d<\frac{L\lambda }{2n_{0}}.$$

To eliminate interference from higher-order diffraction on filtering performance, the grating is engineered to operate exclusively in the zero-order diffraction regime. For compatibility with the incident wavelength range required for demodulation, the period of the all-dielectric grating must satisfy the condition:$\;d<\lambda /(2n_{0})$.

**Figure 2:**
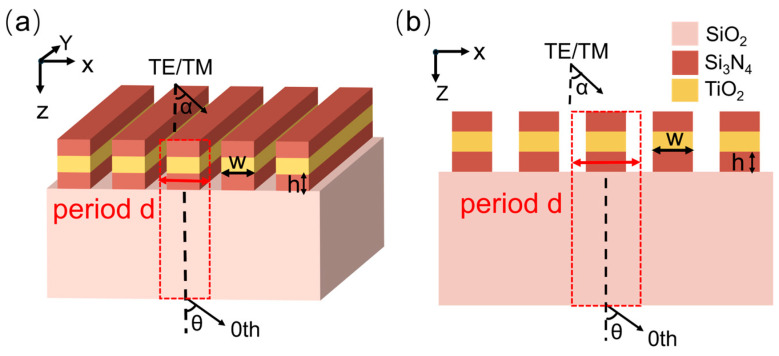
(**a**) Two-dimensional structure diagram of metasurface grating. (**b**) Three-dimensional structure diagram.

The design and simulation of the grating are implemented using the finite element analysis software COMSOL Multiphysics 5.6. The frequency domain electromagnetic waves interface from the wave optics module was employed as the computational physics field. Periodic boundary conditions were implemented to account for the periodic nature of the grating configuration. The subwavelength grating-based edge filter proposed in this work adopts a three-layer rectangular all-dielectric metasurface architecture fabricated on a fused silica substrate. Figure 2a presents a cross-sectional view (2D schematic) of the device, while Figure 2b shows its perspective 3D schematic. The top and bottom layers of the grating ridges are composed of silicon nitride (Si_3_N_4_), while the middle layer consists of titanium dioxide (TiO_2_). The grating grooves are filled with air, which has a refractive index of 1.00. The thickness of each layer of the grating ridges is h=200 nm, the ridge width is w=400 nm, and the grating period is d=680 nm. Incident light is introduced at an angle α=47°.

This study employs the finite element method to numerically calculate the electromagnetic distribution of the designed periodic grating structure. The time-harmonic Maxwell’s equation is solved under periodic boundary conditions:

(3) $$\nabla \times (\nabla \times E)-k_{0}^{2}\epsilon_{r}E=0,$$

where $\epsilon_{r}={\left(n+ik\right)}^{2}$, n and k are the real and imaginary parts of the complex refractive index of the material. To account for material dispersion effects, the complex refractive indices of Si_3_N_4_ and TiO_2_ were derived from experimental datasets reported by Luke et al. [23] and Siefke et al. [24], respectively. The silicon dioxide (SiO_2_) substrate was assigned a fixed refractive index of 1.466 [25]. Through numerical solutions of electromagnetic field vectors at the input and output ports, the zero-order diffraction efficiency (encompassing both transmittance and reflectance) of the grating structure was determined.

## 3. Results and Discussion

### 3.1. Design of the Subwavelength Grating Edge Filter

**Figure 3:**
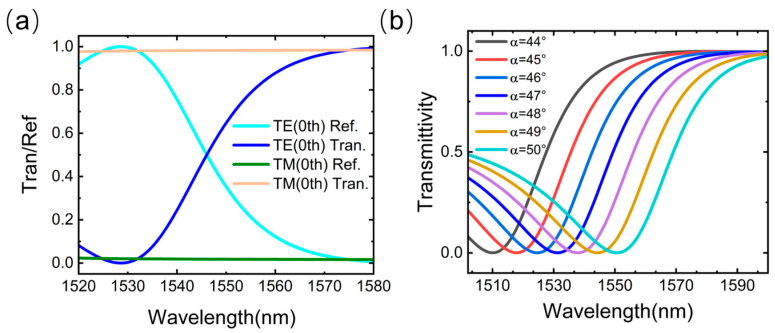
(**a**) Transmittance and reflectance of the super-surface grating for different wavelengths of light. (**b**) Effect of the change of incident angle on the transmittance.

As demonstrated in Figure 3a, at an incident angle of α=47°, the wavelength-dependent transmittance and reflectance of the designed subwavelength grating exhibit distinct behaviors for transverse electric (TE) and transverse magnetic (TM) modes. It can be observed that over the range of 1530–1570 nm: For the TE mode, the transmittance of the grating increases linearly with wavelength, while the reflectance decreases in a complementary linear fashion. For the TM mode, the grating achieves nearly full transmission. To minimize measurement errors and enhance sensitivity, the system incorporates a polarizer to selectively propagate TE-mode light. Figure 3b reveals that the angular tuning of the incident light induces a spectral shift in the grating’s transmission curve. This angle-dependent tunability enables dynamic adjustment of the filter’s operational wavelength range, significantly enhancing its compatibility for practical applications.

**Figure 4:**
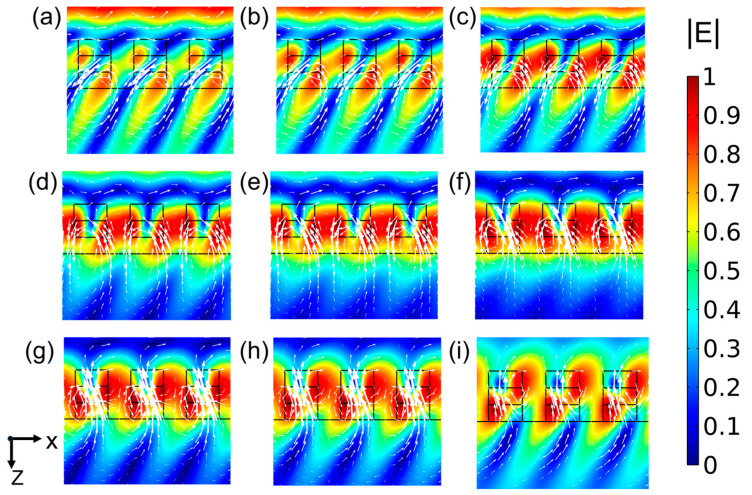
Distribution of the TE-polarized electromagnetic field (white arrows indicate the direction of the magnetic field), corresponding to the wavelength of the incident light 1500 nm (**a**), 1515 nm (**b**), 1530 nm (**c**), 1545 nm (**d**), 1550 nm (**e**), 1560 nm (**f**), 1570 nm (**g**), 1575 nm (**h**), 1590 nm (**i**).

To reveal the electromagnetic mechanisms governing the edge-filtering behavior of the all-dielectric grating, we analyzed TE-polarized electromagnetic field distribution modes in the *xz*-plane across varying incident wavelengths. As shown in Figure 4, the electromagnetic field polarization varies with the incident wavelength. Specifically, at an incident wavelength of 1530 nm, electromagnetic energy localizes predominantly within the air gaps between grating ridges (Figure 4c), corresponding to the minimum transmittance of the grating. This energy concentration can be attributed to guided-mode resonance. As the wavelength increases beyond 1530 nm (Figure 4d–i), the localized electromagnetic field gradually diminishes and shifts toward the top corners of the grating ridges. Concurrently, a portion of the electromagnetic field energy leaks through, and due to wavevector mismatch as the wavelength increases, the transmittance rises with the incident wavelength. The coupling mode evolves with the change of wavelength, endowing the grating with unique transmission and reflection properties, which form the basis for realizing edge filtering.

**Figure 5:**
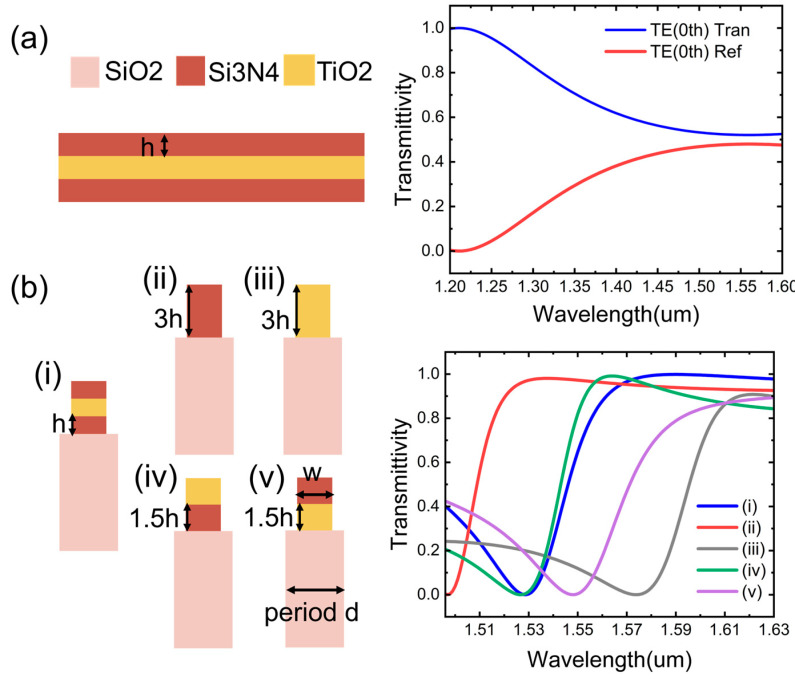
(**a**) A scenario involving three layers of flat materials without a grating structure and its transmittance and reflectance; (**b**) transmittance corresponding to different grating layer structures (**i**) three-layer structure with two layers of Si_3_N_4_ sandwiched by one layer of TiO_2_; (**ii**) single-layer structure with only one layer of Si_3_N_4_; (**iii**) single-layer structure with only one layer of TiO_2_; (**iv**) two-layer structure with Si_3_N_4_ underneath; (**v**) two-layer structure with TiO_2_ underneath.

Considering the complexity of the grating fabrication process, we attempted to simplify the structure of the edge filter device. First, we conducted simulations under consistent conditions for a three-layer film structure without a grating configuration, as shown in Figure 5a. Although the transmittance and reflectance of the three-layer film structure exhibited edge-filtering characteristics across a wide wavelength range, the amplitudes of both were reduced by half, making it difficult to achieve high-quality edge filtering. This highlights the necessity of introducing a grating structure. Additionally, we compared the filtering performance of grating structures with different layer configurations, as shown in Figure 5b. Compared with single-layer and double-layer gratings, the triple-layer grating demonstrated a wider edge-filtering characteristic and the largest amplitude variation in transmittance. Moreover, unlike other structures, the transmittance of the triple-layer grating did not experience a significant downward shift after reaching its peak. These characteristics are sufficient to achieve wide-range, high-quality edge filtering, thereby indicating the necessity of adopting a triple-layer grating structure.

**Figure 6:**
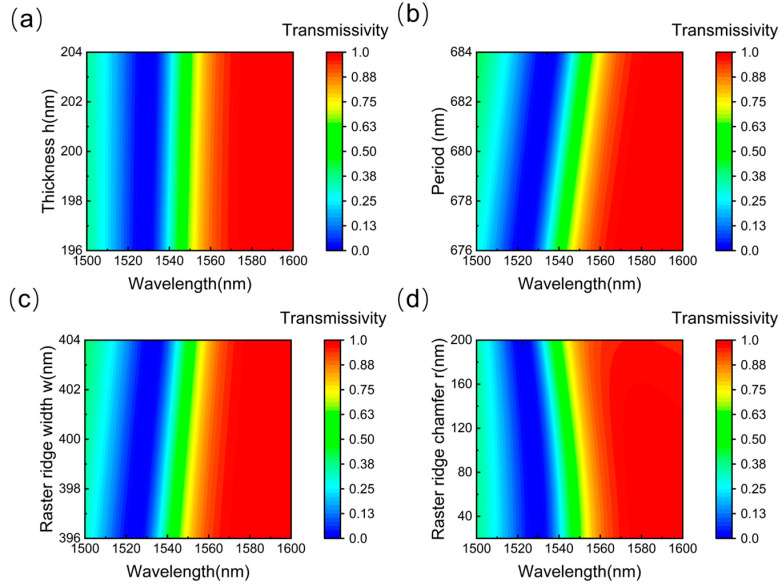
(**a**) The effect of grating layer height variations on transmittance. (**b**) The effect of grating period variations on transmittance. (**c**) The effect of grating ridge width variations on transmittance. (**d**) The effect of grating ridge chamfer variations on transmittance.

To evaluate the performance of the grating, a comprehensive and detailed analysis of its structural parameters is essential. The manufacturing tolerances of the grating must also be meticulously measured, as they hold practical and significant implications for the variations in grating model performance throughout the production process due to manufacturing defects. Herein, the manufacturing tolerances of the proposed grating structure are investigated. As illustrated in Figure 6a–c, the thickness of the grating layer has the least influence on the transmission spectrum. In contrast, an increase in the grating period and ridge width results in a red shift of the refractive index curve, with the grating period exerting the most significant impact. Thus, the operational range of the demodulation system can be tuned by controlling variations in the grating period. Figure 6d illustrates the impact of increasing the ridge chamfer on transmittance. It is evident that when the chamfer radius is less than 100 nm, the impact on grating performance is negligible, indicating that the fabrication process permits reasonable manufacturing tolerances.

### 3.2. FBG Demodulation Based on Subwavelength Grating Edge Filters

When a broadband light source is introduced into an FBG, the reflected Bragg wavelength ($\lambda_{B}$) is determined by the Bragg equation [26]:

(4) $$\lambda_{B}=2n_{eff}\;\Lambda ,$$

where$\;n_{eff}$ is the effective refractive index, Λ is the period of FBG. The Bragg wavelength is related to the grating period and the effective refractive index of the fiber core, both of which are influenced by physical parameters such as strain and temperature. When the temperature varies (ΔT), the grating period and effective refractive index of the FBG are altered, consequently inducing a shift in the Bragg wavelength ($\Delta \lambda_{B}$). The relationship between Bragg wavelength shift and the temperature change can be expressed as [8]:

(5) $$\frac{\Delta \lambda_{B}}{\Delta T}=2n_{eff}\frac{\partial \Lambda }{\partial T}+2\Lambda \frac{\partial n_{eff}}{\partial T}.$$

Substituting the values of $2n_{eff}$ and 2Λ from Equation (4) into Equation (5) yields:

(6) $$\frac{\Delta \lambda_{B}}{\Delta T}=\lambda_{B}\left(\frac{1}{\Lambda }\frac{\partial \Lambda }{\partial T}+\frac{1}{n_{eff}}\frac{\partial n_{eff}}{\partial T}\right).$$

Let $\alpha =\frac{1}{\Lambda }\frac{\partial \Lambda }{\partial T}$, $\xi =\frac{1}{n_{eff}}\frac{\partial n_{eff}}{\partial T}$, where α represents the thermal expansion coefficient and ξ represents the thermo-optic coefficient. Simplifying Equation (6) gives:

(7) $$\Delta \lambda_{B}=\lambda_{B}\left(\alpha +\xi \right)\Delta T.$$

From Equation (7), it can be seen that when α and ξ remain unchanged with the temperature, the temperature change is linearly related to the shift in the Bragg wavelength. By measuring the change in the Bragg wavelength, the corresponding environmental temperature change can be deduced. This constitutes the fundamental principle of FBG temperature sensing. For sapphire FBGs, the Bragg wavelength varies nearly linearly with temperature over a large range [6], and errors can be corrected through signal compensation to reflect the actual temperature changes.

According to coupled-mode theory, the theoretical reflection spectrum of the FBG can be expressed as [27]:

(8) $$f\left(\lambda \right)=\frac{\kappa^{2}{sinh}^{2}(sL)}{{\delta^{2}{sinh}^{2}(sL)+s}^{2}{cosh}^{2}(sL)}\;,$$

where L is the grating length, $\kappa =\frac{\pi }{\lambda }\Delta n_{eff}$ is the coupling coefficient, $\Delta n_{eff}$ is the effective refractive index modulation depth, $\delta =\beta -\frac{2\pi n_{eff}}{\lambda_{B}}$ is the detuning parameter, $\beta =\frac{2\pi }{\lambda }\;n_{eff}$ is the propagation constant, $n_{eff}$ is the effective refractive index, and $s=\sqrt{\kappa^{2}-\delta^{2}}$.

Using the designed subwavelength grating as an edge filter, the Bragg wavelength shift of the FBG is effectively transformed into a variation in optical intensity. Leveraging the transmission and reflection characteristics of the grating edge filter, dual single-point photodetectors are utilized to capture the rational signal, thereby compensating for fluctuations in the source intensity during measurements. The rational signal is expressed as [15]:

(9) $$R\left(\lambda \right)=\frac{t\left(\lambda \right)-r(\lambda )}{t\left(\lambda \right)+r(\lambda )},$$

where $t\left(\lambda \right)\;$and r(λ) denote the transmittance and reflectance of the designed subwavelength grating, respectively, both of which are functions of wavelength.

After passing through the edge filtering system, the optical intensity rational output as a function of the Bragg wavelength can be given by:

(10) $$I\left(\lambda_{B}\right)=\int_{\lambda_{1}}^{\lambda_{2}}R\left(\lambda \right)f\left(\lambda_{B}-\lambda \right)d\lambda ,\;i.e.,\;I=g(\lambda_{B}).$$

Thus, the inverse relationship between the Bragg wavelength and the rational output intensity can be fitted with a polynomial based on the theoretically calculated values, and can also be corrected according to the actual measured values:

(11) $$\lambda_{B}=g^{-1}\left(I\right)=ployfit\left(m\right),$$

where m denotes the order of the polynomial employed. Based on the above principles, this study designs a demodulation system utilizing the subwavelength grating edge filter. The system is characterized by the following features: The FBG-reflected light is an incident in the TM mode through a polarizer at an angle α=47°. After passing through the subwavelength grating with edge filtering effects, the transmitted and reflected light components are converted into equivalent voltage signals by photodetectors PD1 and PD2, respectively. The actual rational output can be obtained as:

(12) $$Rational\;Output=\frac{{PD}_{1}-{PD}_{2}}{{PD}_{2}+{PD}_{1}}.$$

For FBGs with different parameter specifications, their temperature responses also vary. By integrating the transmission and reflection spectra of these FBGs with the curve of the edge filter, the demodulation performance of the designed edge filtering system can be simulated. For sapphire FBGs, it is assumed that the Bragg wavelength varies linearly over a large temperature range. The rational output can be calculated using the transmitted and reflected light intensities obtained from PD1 and PD2, respectively. Consequently, based on the inverse relationship described in Equation (11), the Bragg wavelength of the FBG can be demodulated in real time, thereby determining the corresponding temperature.

**Figure 7:**
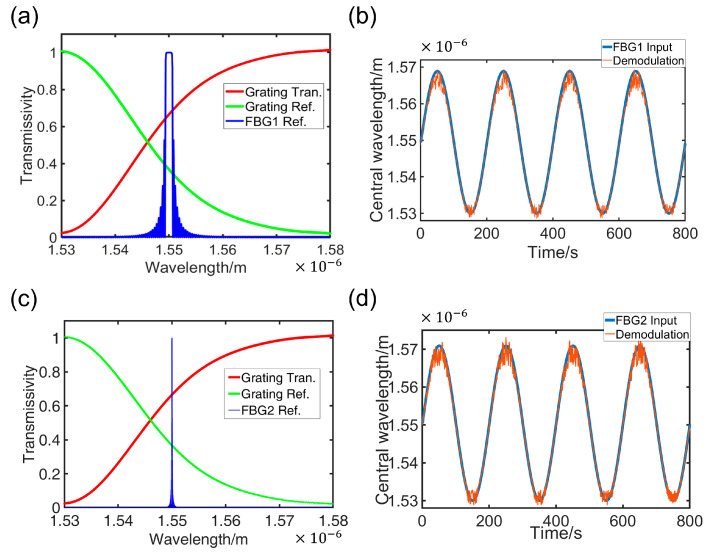
Simulation results of FBG demodulation. (**a**) Spectral reflection of FBG1; (**b**) the true Bragg wavelength and the demodulated signal of FBG1 variation over time; (**c**) spectral reflection of FBG2; (**d**) the true Bragg wavelength and the demodulated signal of FBG2 variation over time.

In this paper, numerical simulation was conducted using different FBGs. The simulation parameters were configured as follows: grating period Λ=537 nm and fiber effective refractive index $n_{eff}=1.445$. Through systematic parameter variation in grating length (L) and effective refractive index modulation depth ($\Delta n_{eff}$), two distinct FBG reflection spectra with customized full-width at half-maximum (FWHM) characteristics were obtained: FBG1: L=2 mm, $\Delta n_{eff}=1.2\times 10^{-3}$, FBG2: L=20 mm,$\;\Delta n_{eff}=1.2\times 10^{-4}$. These differences manifest primarily in the bandwidths of the FBGs, as illustrated in Figure 7a,c.

Here, a sinusoidal variation in the Bragg wavelength is modeled as:

(13) $$\Delta \lambda_{B}=Asin\left(\omega t\right)+\lambda_{0},$$

where A is the amplitude of the signal, $\lambda_{0}\;$= 1550 nm, and the random gaussian noise with signal-to-noise ratio (SNR) of 13 dB is introduced during the demodulation process. In Figure 7b,d, the blue curve represents the true Bragg wavelength variation over time, while the red curve depicts the demodulated signal derived from the inverse relationship. The presence of spikes and fluctuations in the demodulated signal indicates the influence of noise, but this impact on the accuracy of demodulation is relatively minor. Notably, the FBG with a wider bandwidth exhibits greater resilience to noise. The nearly accurate demodulation results obtained for different FBGs demonstrate the feasibility and broad applicability of this demodulation method.

As shown in Table 1, several major edge filtering schemes are compared with the proposed method. The edge filtering demodulation methods that convert FBG wavelength shifts into intensity variations achieve high demodulation speeds. However, their narrow demodulation ranges restrict their applicability to large-range temperature sensing applications. In contrast, the proposed all-dielectric subwavelength grating edge filter achieves wide-range dynamic real-time detection and integrates seamlessly with existing spectrometer optical systems, making it ideal for FBG temperature signal demodulation. Furthermore, the system’s operational range can be dynamically adjusted by modifying the grating’s incident angle, offering exceptional flexibility for practical implementations. On the other hand, the fabrication process for the proposed all-dielectric subwavelength grating linear filter is well established and highly controllable, ensuring the feasibility and reliability of the designed grating in practical applications.

## 4. Conclusions

In this paper, we propose a novel real-time demodulation approach for Fiber Bragg Grating sensing signals employing all-dielectric subwavelength grating edge filters. This study integrates the unique transmission-reflection properties of dielectric nanostructured gratings to achieve real-time demodulation of large-range temperature variation signals in sapphire FBG sensors. Numerical simulation results demonstrate that this demodulation method achieves a wide bandwidth of 40 nm. Furthermore, the demodulation range can be flexibly adjusted by modifying the incident angle and grating parameters, thereby significantly enhancing its practical applicability. The proposed all-dielectric metasurface grating-based edge filter represents a novel approach to FBG demodulation. This method exhibits exceptional compatibility with existing nanofabrication techniques, thereby unlocking substantial potential for practical applications in large-range temperature sensing under extreme thermal-pressure conditions.

## Figures and Tables

**Table 1:** Comparison of the relevant parameters of several edge filtering schemes.

Edge Filter	Reference	Demodulation Range	Demodulation Rate	Linearity
Subwavelength Gratings	This Work	40 nm	Real time	Medium high
WDM	[10]	10 nm	High speed	Medium high
AWG	[11]	2 nm	High speed	High
	[12]	0.3 nm	High speed	High
F-P	[13]	6 nm	5 Khz	Low
Matched FBG	[14]	0.06 nm	500 Khz	Medium
LPFG	[15]	8 nm	NA	High
CFBG	[16]	0.4 nm	NA	High

## Data Availability

Data are contained within the article.
